# Klotho Inhibits Interleukin-8 Secretion from Cystic Fibrosis Airway Epithelia

**DOI:** 10.1038/s41598-017-14811-0

**Published:** 2017-10-30

**Authors:** Stefanie Krick, Nathalie Baumlin, Sheyla Paredes Aller, Carolina Aguiar, Alexander Grabner, Juliette Sailland, Eliana Mendes, Andreas Schmid, Lixin Qi, Nicolae V. David, Patrick Geraghty, Gwendalyn King, Susan E. Birket, Steven M. Rowe, Christian Faul, Matthias Salathe

**Affiliations:** 1 Division of Pulmonary, Allergy, Critical Care and Sleep Medicine, Department of Medicine, University of Miami Leonard M. Miller School of Medicine, Miami, FL 33136 USA; 2 Division of Pulmonary, Allergy and Critical Care Medicine, Department of Medicine, The University of Alabama at Birmingham, Birmingham, AL 35294 USA; 3 Division of Nephrology, Department of Medicine, Duke University School of Medicine, Durham, NC 27710 USA; 4 Division of Nephrology and Hypertension, Department of Medicine and Center for Translational Metabolism and Health, Institute for Public Health and Medicine, Northwestern University Feinberg School of Medicine, Chicago, IL USA; 5 Division of Pulmonary and Critical Care Medicine, Department of Medicine, State University of New York Downstate Medical Center, Brooklyn, NY USA; 6 Department of Neurobiology, The University of Alabama at Birmingham, Birmingham, AL USA; 7 Division of Nephrology and Hypertension, Department of Medicine, The University of Alabama at Birmingham, Birmingham, AL 35294 USA

## Abstract

Chronic inflammation is a hallmark of cystic fibrosis (CF) and associated with increased production of transforming growth factor (TGF) β and interleukin (IL)-8. α-klotho (KL), a transmembrane or soluble protein, functions as a co-receptor for Fibroblast Growth Factor (FGF) 23, a known pro-inflammatory, prognostic marker in chronic kidney disease. KL is downregulated in airways from COPD patients. We hypothesized that both KL and FGF23 signaling modulate TGF β-induced IL-8 secretion in CF bronchial epithelia. Thus, FGF23 and soluble KL levels were measured in plasma from 48 CF patients and in primary CF bronchial epithelial cells (CF-HBEC). CF patients showed increased FGF23 plasma levels, but KL levels were not different. In CF-HBEC, TGF-β increased KL secretion and upregulated FGF receptor (FGFR) 1. Despite increases in KL, TGF-β also increased IL-8 secretion via activation of FGFR1 and Smad 3 signaling. However, KL excess via overexpression or supplementation decreased IL-8 secretion by inhibiting Smad 3 phosphorylation. Here, we identify a novel signaling pathway contributing to IL-8 secretion in the CF bronchial epithelium with KL functioning as an endocrine and local anti-inflammatory mediator that antagonizes pro-inflammatory actions of FGF23 and TGF-β.

## Introduction

Cystic fibrosis is the most common genetic disease in the Western world which decreases life expectancy due to respiratory failure as a result of chronic pulmonary infection and inflammation^1^. A genetically defective cystic fibrosis transmembrane conductance regulator (CFTR) leads to mucociliary dysfunction, increased bacterial colonization and inflammation^1^ that may be even endogenous, based on observations of inflammation preceding infection in CF neonates and young children^2–5^, where bronchoalveolar lavage fluid showed increased levels of IL-8 even in the absence of bacterial infection^3,6^. TGF-β has been characterized as a modifier gene in CF^7^: It is well known that a high-producer TGF-β genotype is associated with more severe lung disease in CF^8,9^. Furthermore, TGF-β levels were elevated in bronchoalveolar lavage fluid from CF patients as well as in conditioned media of CF cells^10,11^, which impaired the therapeutic effect of mutant CFTR correctors and modifiers in CF human bronchial epithelial (CF-HBE) cells^12,13^.

α Klotho (KL) exists as a transmembrane protein and has been characterized as a co-receptor for FGF23 thereby mediating parathyroid hormone secretion and regulating phosphate secretion in the kidney^14,15^. However, it also occurs in a soluble, circulating form, either generated by alternative splicing or by proteolytic cleavage^16,17^. Circulating KL has been described to exhibit anti-inflammatory, anti-fibrotic and anti-senescence effects^18,19^. Mice deficient in KL have a decreased life span and develop lung emphysema consistent with aging^20^.

FGF23 is a member of the fibroblast growth factor family. Mainly secreted by osteocytes, FGF23 regulates mineral metabolism and phosphate homeostasis^21^. In the kidney and the parathyroid gland, FGF23 binds to FGF receptor (FGFR) 1 – α-klotho (KL) complexes, thereby activating the Ras/mitogen-activated protein kinase (MAPK) signaling cascade^22^. In chronic kidney disease (CKD), a state of FG23 excess, FGF23 can also bind to FGFR4, independently of KL. FGF23 mediated activation of FGFR4 has been shown to induce left ventricular hypertrophy and hepatic inflammation through a direct FGFR/phospholipase Cγ (PLCγ)/calcineurin/ and nuclear factor of activated T-cells (NFAT)-dependent mechanism^23,24^.

To date, little is known about KL and FGFR signaling in CF, except that FGF2 might play a key role in tissue regeneration and activation of TGF-β in endothelial cells^25^. In the current study, we analyzed soluble KL and FGF23 levels in plasma from CF patients and attempted to correlate these results with exacerbation status and lung function. We also investigated FGF23 and KL-mediated signaling in CF epithelia and its effect on TGF-β-induced inflammation.

## Results

### TGF-β-induces upregulation of KL in CF-HBEC

**Figure 1:**
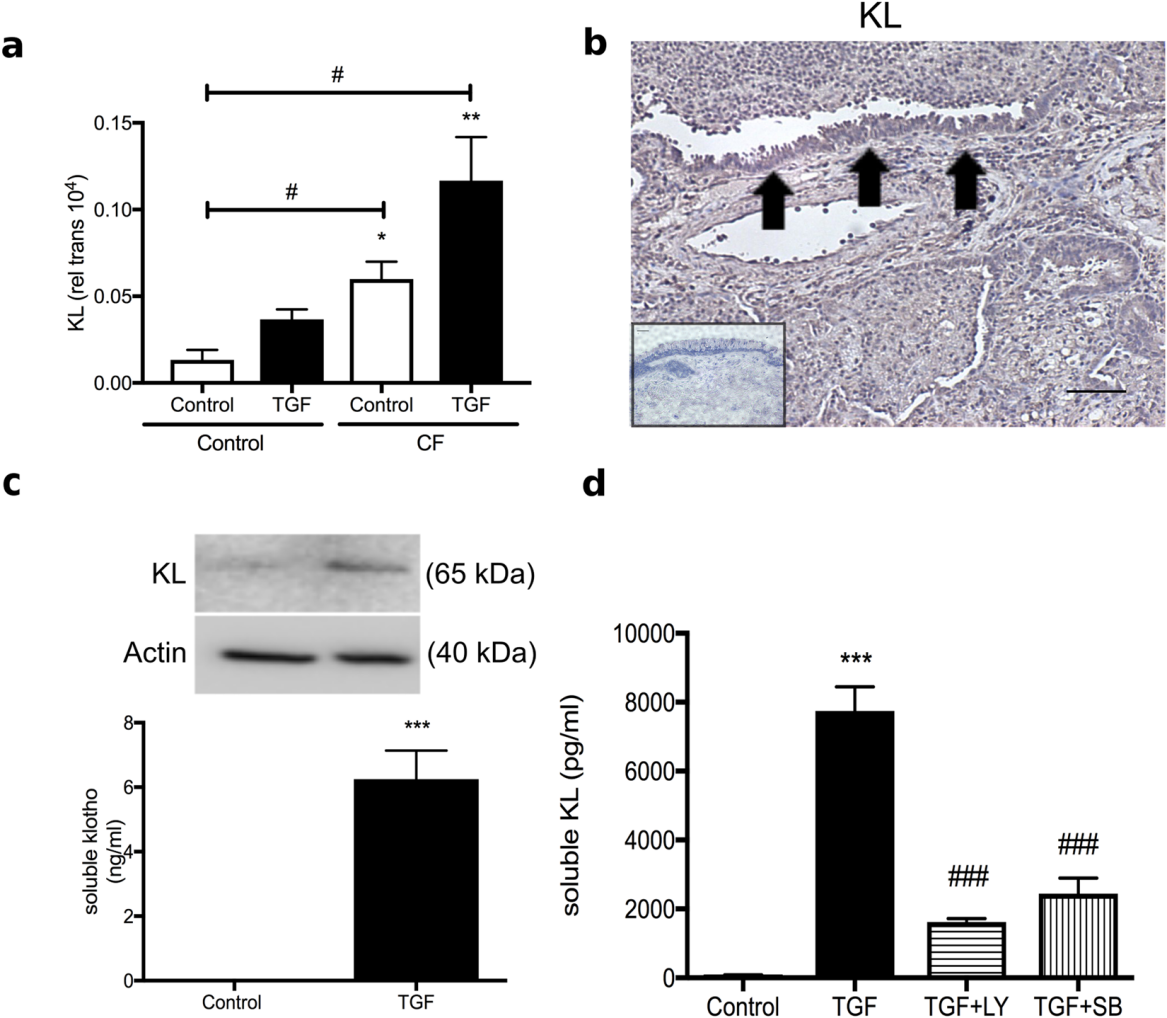
Regulation of KL secretion by TGF-β. (**a**) Bar graphs showing increased KL mRNA levels in CF-HBECs compared to control HBECs from non-smokers. In cultures from both groups, an increase in KL is seen after treatment with TGF-β (10 ng/ml for 24 hours). (**b**) Immunohistochemistry for ΚL localized in the bronchial epithelium (arrows), counterstained with H&E of whole lung tissue from one representative CF patient, compared to a negative control (secondary antibody omitted, lower left corner, 20X magnification). Scale bar is 40 μm. (**c)** Upper part: Immunoblot analysis of KL protein levels from CF-HBECs (representative blot from 3 different CF lungs), treated with either vehicle or TGF-β (10 ng/mL) for 24 hours. The full-length blots are presented in Supplementary Figure 1. Lower part: Assessment of soluble KL levels by ELISA from basolateral CF-HBEC media with and without TGF-β stimulation. (**d**) Secreted soluble KL levels of CF-HBECs (in ng/ml) exposed to TGF-β (10 ng/mL) for 24 h and pretreated with LY2157299 (10 μM) or SB203580 (10 μM). All n = 3 independent experiments from 3 different lungs showing mean ± S.E. with ***P < 0.005 compared to control and ^####^P < 0.005 compared to TGF-β treatment.

Using quantitative real time PCR, we assessed FGF23 and KL expression in primary CF human bronchial epithelial cells, cultured at the air liquid interface (ALI). mRNA expression of FGF23 was not found by PCR in CF-HBECs >40 cycles; data not shown) but KL mRNA was detectable with levels being higher in CF-HBECs compared to HBECs from control patients without lung disease (Fig. 1a). In addition, KL mRNA increased significantly when CF-HBECs were stimulated with TGF-β for 24 hours (Fig. 1a). Next, KL protein expression was analyzed by immunohistochemistry. Paraffin embedded slides from CF lung tissue, stained with anti-rabbit KL antibody and counterstained with hematoxylin-eosin, demonstrated remodeled lung architecture and inflammatory infiltrates (Fig. 1b). Compared to a negative control (primary antibody omitted; smaller image lower left corner), immunostaining with a KL antibody^26^ revealed a signal localized to the partially destroyed bronchial epithelium (Fig. 1b, arrows). CF-HBECs, stimulated with TGF-β for 24 hours, also showed an increase of KL protein levels in whole cell lysates and secretion of soluble KL into the basolateral media from intact cells (Fig. 1c). We did not detect any apical secretion of KL (data not shown).

### TGF-β induced upregulation of KL depends on TGF-β receptor and p38 signaling

To understand which signaling pathways are involved in upregulating KL by TGF-β, CF-HBECs were preincubated with LY2157299, a TGF-β receptor 1 inhibitor, or SB203580, a p38 MAPK inhibitor. Both blunted the TGF-β-induced upregulation of KL protein secretion (Fig. 1d).

### TGF-β upregulates FGFR 1 expression in CF-HBEC

**Figure 2:**
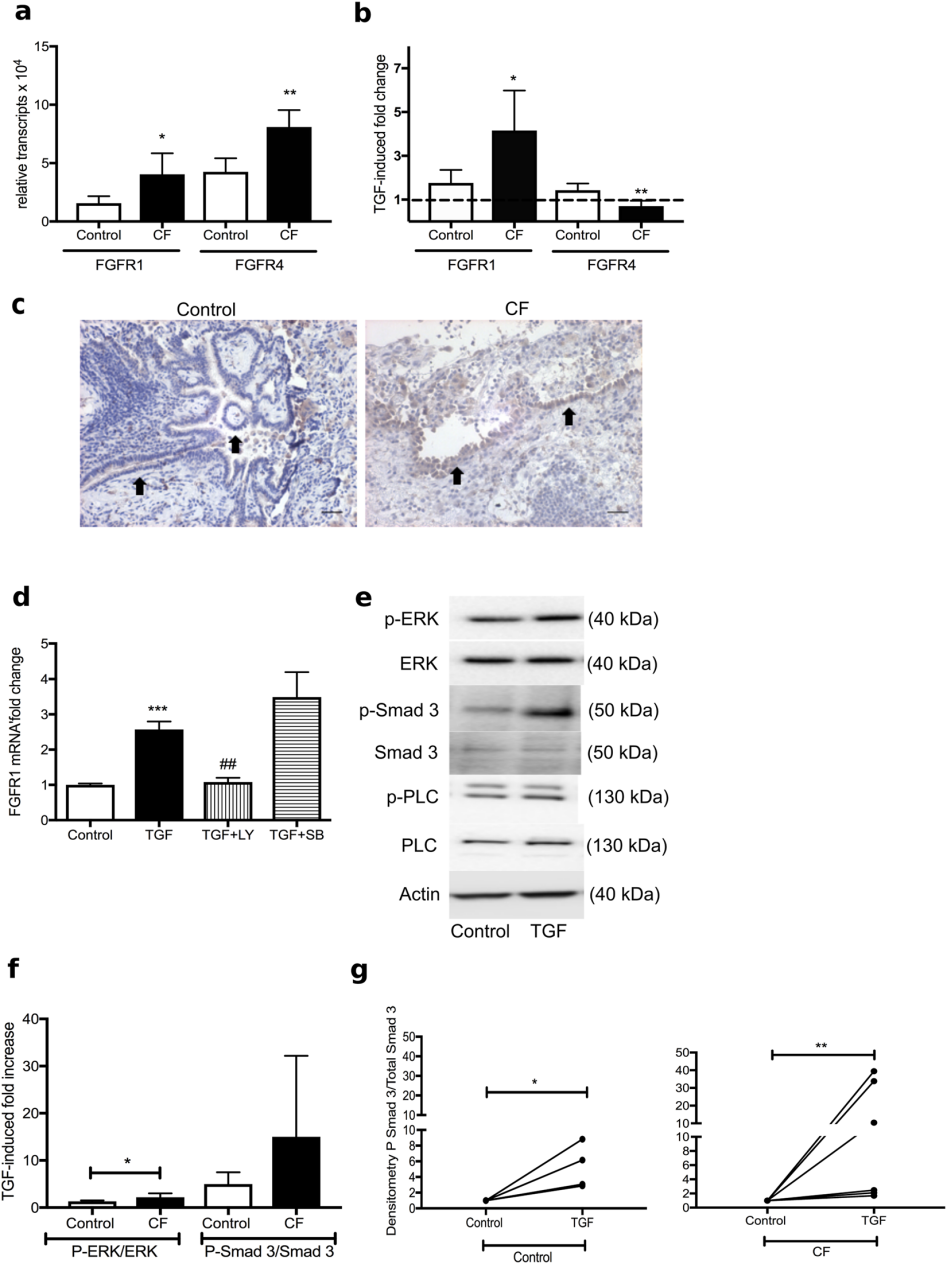
TGF-β induced upregulation of FGFR1 in CF-HBECs. (**a**) FGFR1 and FGFR4 mRNA levels in HBECs from nonsmokers and CF patients at baseline and (**b**) after stimulation with TGF-β (10 ng/mL) for 24 hours. (**c**) Immunohistochemistry staining with anti-FGFR1-HRP and hematoxylin eosin counterstain of whole lung sections from one control patient (non-smoker) compared to a CF patient (20X, scale bars are 40 μm): staining of the bronchial epithelium (arrows) was detected and increased in the CF lung section (representative specimen from a total of 4 lungs in each group). (**d**) FGFR1 mRNA levels of CF-HBECs, exposed to TGF-β for 24 hours (10 ng/mL) ± LY2157299 (10 μM) or SB203580 (10 μM). (**e**) Representative immunoblots are shown after stimulation of CF-HBEC with TGF-β (30–90 min, 10 ng/ml): increased Smad 3 and increased ERK phosphorylation were seen without changes in PLCγ phosphorylation. (**f**) Bar graphs showing densitometric analysis of TGF-β induced fold changes in phospho ERK/total ERK and phospho Smad 3/total Smad 3 ratios in control HBECs (n = 5) compared to CF-HBEC (n = 6). (**g**) Dot plot indicating densitometric changes in phospho Smad/ total Smad 3 ratios in HBECs and CF-HBECs when untreated and TGF-β treated, shown as fold change increase using unpaired t-test and Mann-Whitney post test. Data is presented as means ± S.E. with *P < 0.05, **P < 0.01, ***P < 0.001 and ^##^P < 0.01 compared to TGF-β treatment. The full-length blots are presented in Supplementary Figure 2.

KL is known to be a co-receptor for FGF23 signaling which can occur via different FGF receptors. We assessed FGFR mRNA expression levels in CF-HBECs and control HBECs. mRNA of FGFR1 and FGFR4 was detected with significantly higher levels of both in CF-HBECs (Fig. 2a). Upon TGF-β stimulation, mRNA expression of FGFR1 but not FGFR4 significantly increased in CF-HBECs when compared to unstimulated cells, whereas FGFR4 mRNA was downregulated (Fig. 2b). Immunohistochemical analysis of FGFR1 in lung tissue sections from nonsmoking subjects and CF patients also showed increased signals in CF sections, with localization of FGFR1 to the bronchial epithelium (arrows in Fig. 2c). Preincubation of CF-HBECs with LY2157299 inhibited TGF-β-induced FGFR1 upregulation whereas SB203580 had no effect, indicating that p38 MAPK signaling was not involved in the regulation of FGFR1 mRNA expression (Fig. 2d).

### TGF-β induces phosphorylation of Smad 3 in CF-HBECs

When CF-HBECs were stimulated with TGF-β for 30 min, a mild increase in phosphorylation of ERK, a strong increase in phosphorylation of Smad 3 and no change in phosphorylation of PLCγ were seen (Fig. 2e). In order to assess differences between CF-HBEC and HBEC from control patients without lung disease, we used densitometric analysis of immunoblots and compared ratios of Phospho-ERK/total ERK and Phospho Smad 3/total Smad 3 between these groups. Despite the mild increase in ERK phosphorylation, there was significantly more phospho ERK in CF cells compared to control cells (Fig. 2f). Phospho Smad 3 fold increases in TGF-β treated CF-HBEC were also higher though very variable from 2 fold up to 40 fold (Fig. 2f), though by employing nonparametric analysis with t-test and Mann Whitney post test, both increases in control HBEC (Control) and CF-HBEC (CF) were significant (Fig. 2g).

### Soluble KL levels are unchanged but FGF23 levels are increased in CF patients

**Figure 3:**
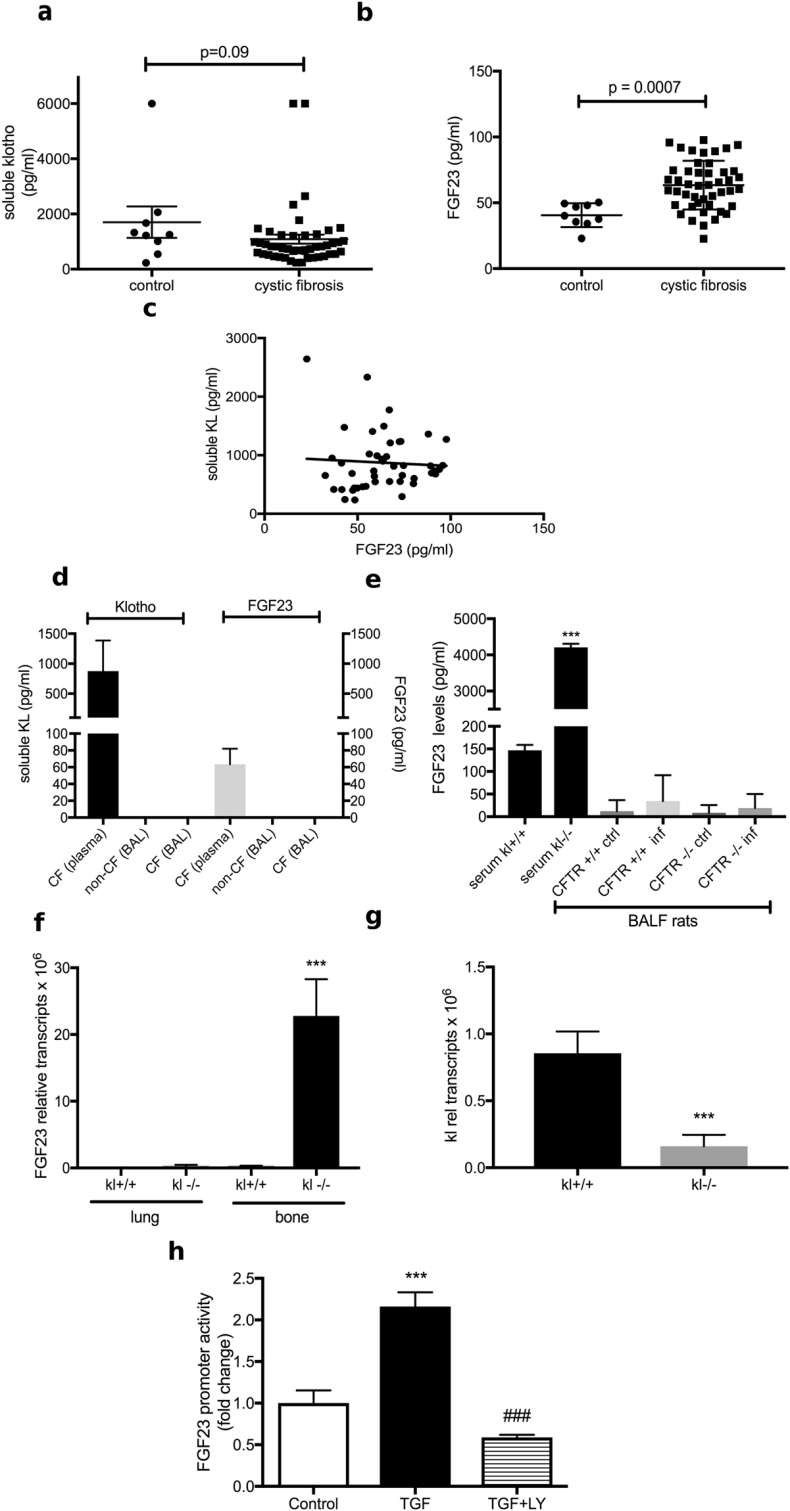
Plasma KL levels are unchanged, but plasma FGF23 levels are increased in CF patients. (**a**) Comparison of soluble KL plasma levels in subjects with and without CF. The control group consisted of 8 subjects without any lung disease (normal FEV1% predicted). (**b**) Assessment of plasma FGF23 levels in the same study population indicating a significant increase in CF patients (p = 0.0007). (**c**) Dot plot showing no significant correlation between FGF23 and KL levels in CF patients. Two outliers were removed that would have made a linear regression positive; however, statistical analysis using runs testing showed that including them made linear regression analysis inappropriate. Statistical analysis was done using Student’s t test showing mean ± S.D. as indicated in appropriate graphs or shown is Pearson correlation coefficient (R) and significance (P). (**d**) Bar graphs indicating soluble KL levels (Klotho) and FGF23 levels (FGF23) in BAL samples from pediatric non-CF versus CF patients in comparison to their respective plasma levels in CF patients. In BAL fluid, both soluble KL and FGF23 could not be detected. (**e**) Bar graphs indicating FGF23 levels in plasma from kl^+/+^ and kl^−/−^ mice. Next to these are BAL samples from CFTR^−/−^ rats and their wild type littermates ± infection with 3 × 10^6^ CFU of a clinical mucoid Pseudomonas (PAM57–15) by intratracheal instillation^27^. BAL was obtained 7 days after infection. (**f**) FGF23 mRNA levels from total lung and bone of kl^+/+^ and kl^−/−^ mice. (**g**) Bar graphs indicating kl mRNA levels from total bone RNA from kl^+/+^ and kl^−/−^ mice. (**h**) Bar graphs showing fold change increases in FGF23 promotor activity in MC3T3-E1 osteoblast precursor cells after stimulation with TGF-β (10 ng/ml) for 24 hours, which was abrogated by pre-incubation with the TGF-β receptor inhibitor LY2157299 (10 μM). (n = 3 independent experiments showing means ± S.E. with ***P < 0.001 compared to control and ^##^P < 0.01 compared to TGF-β treatment).

In order to assess our *in vitro* data for relevance *in vivo*, we measured soluble KL and FGF23 levels in plasma samples from 8 control patients without lung disease and 48 CF individuals. CF patients showed no difference of soluble KL levels (Fig. 3a), but had significantly higher FGF23 plasma levels when compared to patients without lung disease (Fig. 3b; p = 0.0007). There was no correlation between KL and FGF23 levels (Fig. 3c). Furthermore, stratifying the CF patients into exacerbators and non-exacerbators did not reveal any new correlation (data not shown).

### Soluble KL FGF23 levels are not detectable in bronchoalveolar lavage (BAL) fluid

In order to show *in vivo* relevance of KL and FGF23 in the “inflamed CF lung”, we measured both soluble KL and FGF23 in bronchoalveolar lavage fluid from pediatric CF patients (average age 7 ± 5 years) and compared them to age and gender matched non-CF patients. There was neither soluble KL nor FGF23 detectable in the BAL samples (Fig. 3d). Since these samples had been stored for an extensive time, we also analyzed BAL samples from CFTR knockout rats and their littermates under baseline conditions and after infection with 3 × 10^6 CFU of a clinical mucoid Pseudomonas (PAM57-15) by intratracheal instillation^27^. FGF23 levels were measured in comparison to plasma samples from 4 kl^+/+^ and 4 kl^−/−^ mice. While plasma levels of FGF23 were significantly upregulated in the kl^−/−^ and at least detectable in the kl^+/+^ mouse, we were not able to detect any FGF23 protein in the BAL fluid of any of the different groups of rats (Fig. 3e).

*FGF23 secretion by bone and in response to TGF-β*. Since FGF23 and KL were not detectable in BAL fluid, we investigated possible sources of plasma FGF23 elevation in CF lung disease. Since we know from previous reports that the bone is a main source of FGF23^28^, we isolated total RNA from the tibia, lung, liver, heart, kidney and spleen of 4 kl^−/−^ and kl^+/+^ mice. Interestingly, there was no high FGF23 expression in the lung and the other organs, but there was a drastic increase of FGF23 mRNA in the bone from kl^−/−^ mice (Fig. 3f), which do not have consistently detectable KL mRNA (Fig. 3g). Since CF is an inflammatory disease and circulating cytokines such as IL-1β and TNF-α have been shown to increase FGF23 expression in osteocytes^28^, we hypothesized that TGF-β, a known prognostic marker in CF, can activate osseous FGF23 secretion, thereby explaining the elevated FGF23 plasma levels in CF patients. Indeed, MC3T3-E1 osteoblast precursor cells, stimulated with TGF-β for 24 hours significantly induced FGF23 reporter gene activity compared to control cells. This response was inhibited by pre-incubation with the TGF-β receptor inhibitor LY2157299 (Fig. 3h).

### TGF-β and FGF23 induce IL-8 secretion from CF-HBECs in an additive fashion

**Figure 4:**
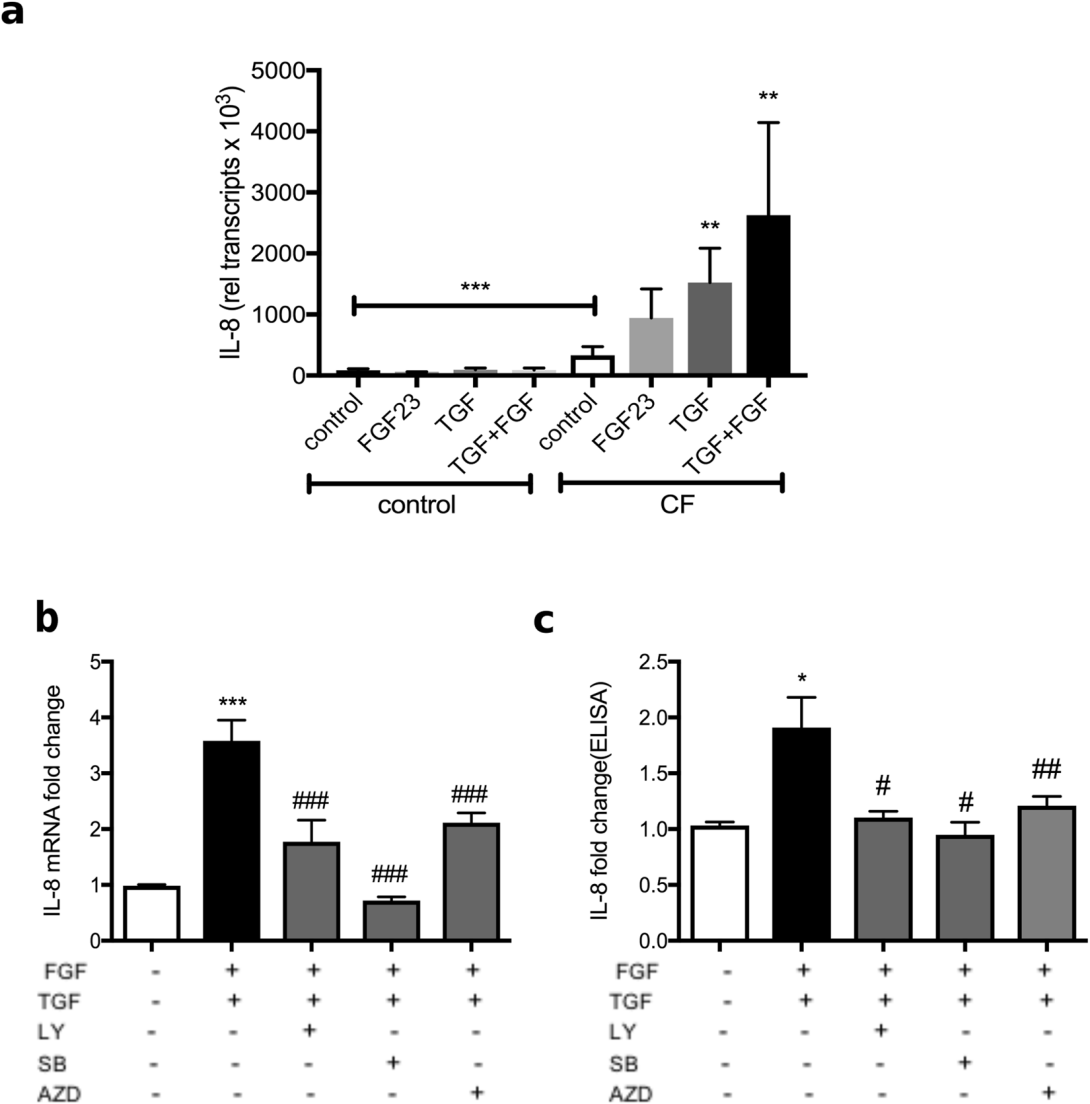
Exposure to TGF-β ± FGF23 increases IL-8 secretion in CF-HBECs. (**a**) Bar graphs indicating relative transcript numbers of IL-8 mRNA, compared to GAPDH, of both HBECs and CF-HBECs after stimulation with FGF23 (25 ng/mL) ± TGF-β (10 ng/mL) for 24 h. (**b**/**c**) Inhibition of either FGF23 (via FGFR1) or TGF-β (via p38) signaling prevented the increase in (**b**) IL-8 mRNA expression and (**c**) IL-8 secretion, suggesting that these pathways are both involved. TGF-β signaling was inhibited by LY2157299 (10 μM), p38 MAPK by SB203580 (10 μM) and FGFR1 signaling by AZD4745 (5 nM). All n = 3 independent experiments from 3 different lungs. In graphs, means ± S.E are shown. *P < 0.05, **P < 0.01, ***P < 0.005 for comparisons with the control and ^###^P < 0.005 for comparison with the treated group.

Since we showed that both FGF23 and TGF-β signaling is activated in CF-HBEC, we treated both HBECs and CF-HBECs with either human recombinant FGF23, TGF-β or both for 24 hours and assessed IL-8 mRNA levels. Already at baseline, CF-HBECs had significantly increased relative transcript numbers of IL-8 compared to control HBECs. Only in CF-HBECs, FGF23, TGF-β and both stimuli together increased IL-8 mRNA significantly (Fig. 4a).

### MAPK p38, FGFR1 and TGF-βR signaling all contribute to TGF-β-induced IL-8 secretion

To further elucidate the signaling pathway involved leading to IL-8 secretion, we treated CF-HBECs with TGF-β and FGF23 and either LY2157299, SB203580, or AZD4547, a FGFR1–3 inhibitor. Blockade of TGF-βR1, p38 and FGFR1–3 all inhibited TGF-β mediated IL-8 mRNA upregulation (Fig. 4b) and IL-8 protein secretion into the basolateral media (Fig. 4c).

### Soluble KL inhibits IL-8 increase in CF-HBECs

**Figure 5:**
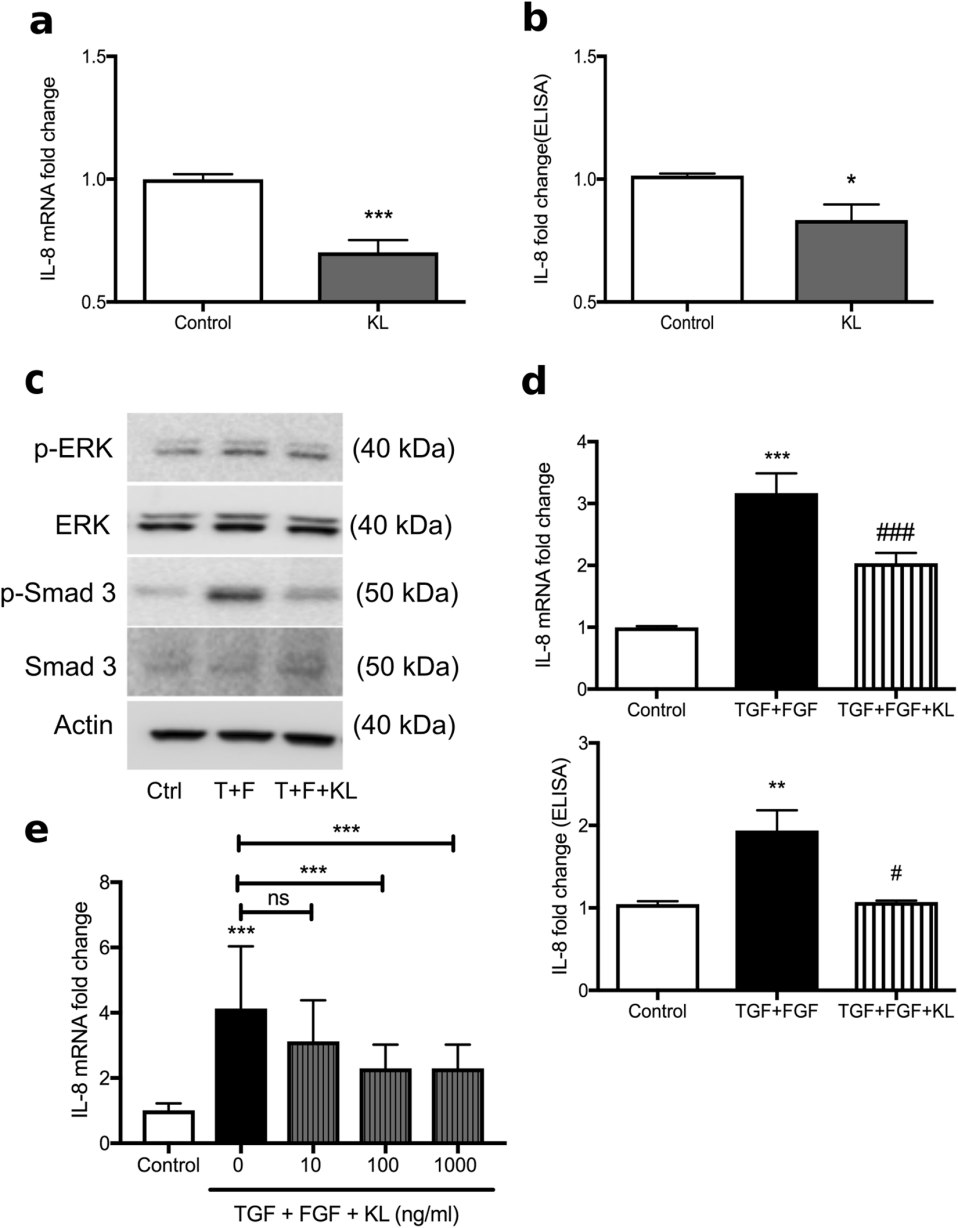
Effect of recombinant human soluble KL on TGF-β ± FGF23-induced inflammation in CF-HBECs. (**a**) A baseline decrease in IL-8 mRNA levels and (**b**) basolateral IL-8 secretion is seen in CF-HBECs after exposure to soluble KL for 24 hours (100 ng/ml). (**c**) Representative immunoblots showing phospho-ERK, total ERK, phospho-Smad 3 and total Smad 3 in lysates from CF-HBECs after stimulation with TGF-β + FGF23 (30 min, 10 and 25 ng/mL, respectively) ± preincubation with soluble KL (100 ng/ml*)*. The full-length blots are presented in Supplementary Figure 2. (**d**) Recombinant soluble KL (100 ng/ml) for 24 hours inhibits TGF-β + FGF23-induced increases of IL-8 mRNA expression and basolateral IL-8 protein secretion from CF-HBECs. (**e**) CF-HBEC were pre-incubated with different concentrations of soluble KL (0, 10 and 100 ng/ml) and TGF-β + FGF23-induced IL-8 mRNA expression was assessed showing only attenuation when KL concentrations ≥ 100 ng/ml were used. All n = 3 independent experiments from 3 different lungs. In graphs, mean ± S.E is shown. *P < 0.05, **P < 0.01, ***P < 0.005 for comparisons with the control and 1–3 ^#^P for comparison with the stimulated group.

Since FGF23 and TGF-β signaling increased IL-8 secretion in CF-HBECs, the role of KL was assessed. CF-HBECs were stimulated with human recombinant KL. This caused a decrease of baseline IL-8 mRNA expression (Fig. 5a) and IL-8 secretion (Fig. 5b). Then, CF-HBECs were stimulated with TGF-β and FGF23 and the role of KL was again assessed in this setting. Incubation with KL did not alter TGF-β + FGF23 induced phosphorylation of ERK but caused a marked decrease in phosphorylation of Smad 3 (Fig. 5c), which ultimately decreased both FGF23 and TGF-β−induced IL-8 responses as indicated by reduced IL-8 mRNA and protein levels (Fig. 5d). Since TGF-β alone could also induce KL secretion (Fig. 1c), it was surprising that induction of endogenous KL (average 6.25 pg/ml) which was not sufficient to dampen IL-8 secretion. Therefore, different concentrations of exogenous KL were used for pretreatment of TGF-β + FGF23 stimulated CF-HBEC. These data show that only higher concentrations of soluble KL (100 ng/ml or 1000 ng/ml) had physiological impact on the TGF-β + FGF23 induced IL-8 secretion (Fig. 5e).

### Overexpression of transmembrane KL inhibits TGF-β and FGF23-induced inflammation in HBECs

**Figure 6:**
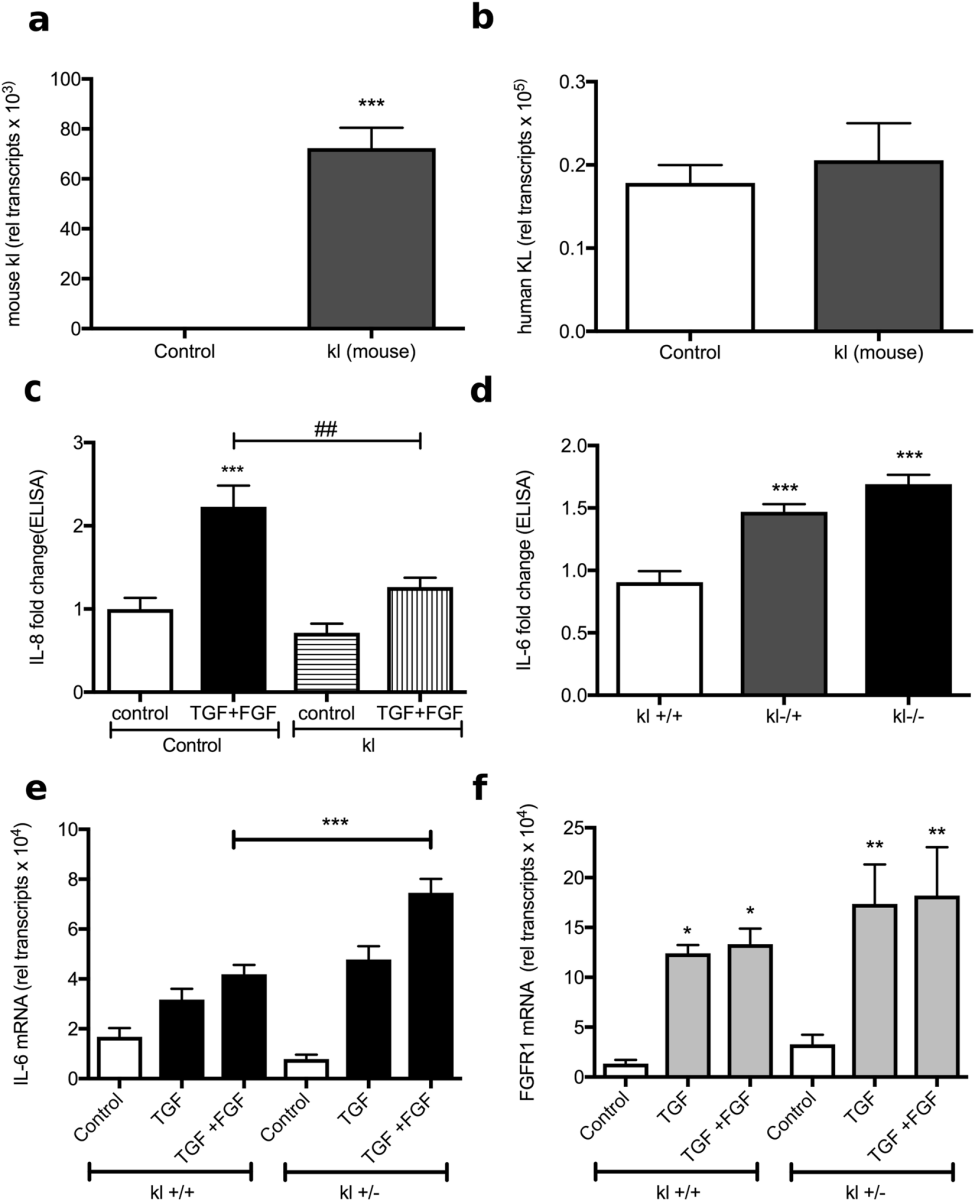
Modification of KL levels changes IL-6 and IL-8 expression. (**a**) Relative transcript numbers of murine kl mRNA and (**b**) endogenous human KL mRNA are shown in fully differentiated HBECs, either control infected or infected with murine full length kl expressing lentiviruses. (**c**) Secreted IL-8 protein levels in both control and murine kl expressing HBECs ± treatment with TGF-β + FGF23 show attenuation of IL-8 secretion in kl infected HBECs. All n = 3 independent experiments from 3 different lungs. (**d**) Secreted IL-6 protein levels at baseline are significantly elevated in murine tracheal epithelial cells (MTECs) isolated from kl^−/−^ and kl^+/−^ mice when compared to wild type controls. Bar graphs indicating (**e**) IL-6 mRNA and (**f**) FGFR1 mRNA from wild type versus kl^+/−^ MTECs after stimulation with TGF-β ± FGF23. MTECs were pooled from 3–4 mouse lungs and 3 independent experiments were performed. All bar graphs are mean ± S.E. *P < 0.05, **P < 0.01 and ***P < 0.005 compared to control.

In order to assess whether the transmembrane form of KL showed a similar effect, HBECs were infected using a lentiviral construct which contained the murine full-length kl. Infected cells showed significantly elevated murine kl mRNA levels when compared to control cells (Fig. 6a). This did not change the expression of endogenous human KL mRNA levels in infected cells (Fig. 6b). To further elucidate the role of KL on IL-8 induced inflammation, we exposed HBECs overexpressing murine kl to TGF-β + FGF23 for 24 hours. Overexpressing murine kl significantly inhibited TGF-β + FGF23-mediated secretion of IL-8 (Fig. 6c).

### Kl deficiency promotes TGF-β and FGF23-induced IL-6 secretion

We isolated murine tracheal epithelial cells (MTEC) from wild-type (kl^+/+^), heterozygous (kl^+/−^) and homozygous klotho hypomorphic mice (kl^−/−^). Since mice lack IL-8 production, we assessed for other pro-inflammatory cytokines. Homozygous as well as heterozygous MTECs exhibited increased baseline IL-6 secretion compared to wild type cells (Fig. 6d). Stimulation with TGF-β  + FGF23 for 24 hours led to a significantly higher increase in IL-6 in the MTECs, heterozygous for kl, when compared to the wild type cells (Fig. 6e). Besides, kl reduction did not alter the TGF-β and FGF23-induced increases in FGFR1 mRNA (Fig. 6f). These data suggest that KL has an anti-inflammatory effect when endogenously expressed, over-expressed or given exogenously, independent of being in its membranous or soluble form. Furthermore, there are no species-specific variations in its anti-inflammatory properties.

## Discussion

KL has been characterized as a protective factor against oxidative stress in lung disease and mice deficient in kl show an aging phenotype including lung emphysema^20,29^. To date, little is known about its role in chronic inflammatory lung diseases and its underlying signaling mechanism^30^.

**Figure 7:**
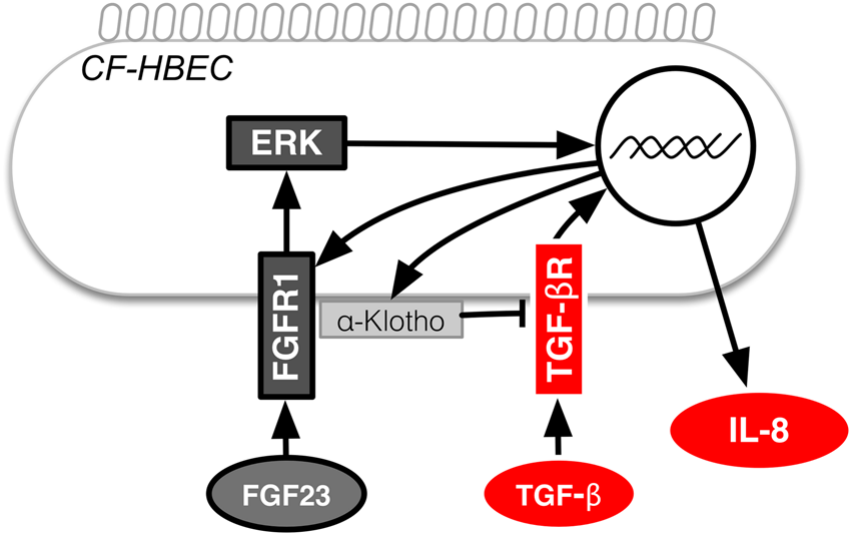
Diagram illustrating the effect of TGF-β and FGF23 on CF bronchial epithelial cells. TGF-β upregulates FGFR1 and together with FGF23, they induce IL-8 expression via activation of ERK and TGF-βR signaling. In addition, TGF-β induces KL secretion, which attenuates IL-8 secretion by inhibition of TGF-βR signaling. Definition of abbreviations: IL = interleukin, FGF = fibroblast growth factor, FGFR = FGF receptor, ERK = Extracellular Signal regulated kinases.

This study examined for the first time the role of KL in CF. KL was secreted by the CF bronchial epithelium as a response to TGF-β stimulation and subsequent upregulation of FGFR1 (Fig. 7). In CF patients, we observed a significant elevation of plasma FGF23 levels, for which KL can function as a co-receptor. We also evaluated TGF-β plasma levels in CF patients which did not correlate with either FGF23 or KL levels (data not shown).

KL has been shown to be decreased in COPD airways and after exposure to ozone or cigarette smoke^26^. In our CF patients, soluble plasma KL levels were not different compared to control subjects, in contrast to upregulation in airway epithelial cells when treated with TGF-β. KL may not reach the blood stream despite its upregulation and this augmentation was insufficient to block signaling, in contrast to exogenous excess of KL. Thus, a possible explanation for this discrepancy could be an imbalance of KL production and consumption locally at the bronchial epithelium. At baseline, CF bronchial epithelia try to compensate for elevated FGF23 levels by KL secretion but the CF lung is chronically inflamed, resulting in degradation. Previous reports demonstrated increased proteolytic cleavage due to protease-antiprotease imbalance in the lung^31^. Our data though suggests that the source of FGF23 is not the lung emphasizing systemic involvement in CF associated inflammation. Since it is well known in CF that bone is affected, too, with a lot of CF patients suffering from bone disease^32,33^, our data suggests that TGF-β could be an inducer of osseus FGF23 secretion. KL though seems to be locally secreted. We could clearly find basolateral secretion from CF-HBEC which could work in an autocrine or paracrine manner. The secretion of KL though was not sufficient to raise systemic KL levels in CF and to attenuate systemic inflammation. We did not detect any KL in the BAL fluid. Therefore, local degradation or insufficient production could be an explanation. Further studies are needed to decipher the exact mechanisms involved in KL production and catabolism.

KL signaling has been closely linked to FGF23 signaling with KL being a co-receptor for FGF23 via FGFR1/ERK signaling, which has been described both in kidney and the parathyroid gland^22,34^. Furthermore, FGF23 has been shown to act independently of KL via FGFR4/PLCγ/NFAT signaling that was characterized in the heart and liver^23,35^. Others have shown that KL can bind to TGF-β receptor II in the kidney, ameliorating renal fibrosis^36^.

In contrast to these and our data showing pro-inflammatory actions of TGF-β with inhibitory effects on mucociliary clearance and CFTR function in normal or CF bronchial epithelia^12,37,38^, several other reports demonstrate TGF-β-mediated anti-inflammatory signaling including decreased Smad 3 levels in CF airway cells^39–42^. These discrepancies could be explained by various facts including: 1) dose-dependent effects of TGF-β, 2) differences in cell types (nasal epithelial versus bronchial epithelial cells, cell lines versus primary cultures), primary culture methods (submerged versus air-liquid interface), or 3) disease entity (asthma versus CF). The experiments presented in this study were all done in primary human bronchial epithelial cells from either control or CF patients, differentiated at the air liquid interface. Furthermore, one other study used mouse nasal epithelial cells from CFTR knockout mice^41^. It has been shown repeatedly, however, that these mice are not representative of CF lung disease due to the fact that they do not develop spontaneous bacterial infections typical of CF and their airways can clear large bacterial inocula^43,44^.

Our data points to TGF-β-induced FGF23 signaling via upregulation of FGFR1 leading to IL-8 secretion. KL decreases IL-8 secretion by reducing phosphorylation of Smad 3 rather than via FGFR1/ERK signaling which seems counterintuitive. However, at the same time, KL inhibits TGF-β signaling to reduce expression of IL-8. By either replacing KL or inhibiting FGFR1, IL-8 secretion was reduced. Therefore, both are potential anti-inflammatory strategies in cystic fibrosis. Other anti-inflammatory therapies have not worked well so far due to systemic side effects^45^. FGFR1 inhibitors are currently under development and studied in multiple cancers as treatment options^46–49^. In addition, we characterized FGFR1 as the dominating isoform in the pathophysiology of CF lung disease.

In summary, our data depicts a novel signaling pathway in cystic fibrosis lung disease. We also describe a feedback mechanism between FGF23 and KL as allies as well as opposing mediators, thus introducing new therapeutic options for targeted anti-inflammatory therapy in CF. Future mechanistic *in vitro* and *in vivo* studies are needed to fully elucidate the proposed mechanism.

## Material and Methods

All data generated or analyzed during this study are included in this published article.

### Study Approval

The clinical study was conducted according to the Declaration of Helsinki principles and had been approved by the University of Miami institutional review board. Prisoners were not included in our study. Written informed consent was received from each participant prior to inclusion in the study. Human airways form CF patients were obtained at time of transplant with informed consent approved by the University of Miami institutional review board. Human airways from healthy subjects were obtained from organ donors whose lungs were rejected for transplant. Institutional review board-approved consent for research was obtained by the Life Alliance Organ Recovery Agency of the University of Miami or the LifeCenter Northwest and conformed to the Declaration of Helsinki. Institutional review board approval was also obtained to obtain access to the BAL samples at the University of Alabama at Birmingham. All animal protocols were approved by the Institutional Animal Care and Use Committees at the University of Miami and the University of Alabama at Birmingham and carried out in accordance to their guidelines.

### Study Design

This was a prospective, single-center cohort study approved by University of Miami’s Institutional Review Board, in which 48 CF patients and 8 control patients without lung disease in our center agreed to donate plasma for assessment of FGF23 and soluble KL levels. Blood samples were collected, centrifuged at 1500 rpm for 15 min at 4 °C and plasma was stored at −80 °C for 3–6 months until use. In addition, BAL samples were obtained from the UAB Biorepository of the Cystic Fibrosis Research Center from pediatric 13 non-CF and 15 CF patients. All animal protocols were approved by the Institutional Animal Care and Use Committees at the University of Miami and the University of Alabama at Birmingham.

### Air Liquid Interface (ALI) Cell Culture

Human bronchial epithelial cells from cystic fibrosis patients (CF-HBEC) were isolated and cultured using the ALI model as described previously^38,50^ and with IRB approved protocols. Murine tracheal epithelial cells (MTEC) were obtained from wild type mice and mice (SV129 background), heterozygous and null for the klotho gene^20^. These cells were cultured and differentiated according to an adapted protocol of You *et al*.^51,52^. Cells were fully differentiated after approximately 2–3 weeks and then used for experiments. Additional non-CF human airways were obtained from organ donors whose lungs were rejected for transplant. Institutional review board-approved consent for research was obtained by the Life Alliance Organ Recovery Agency of the University of Miami or the Life Center Northwest.

### Assessment of FGF23 production by osteocytes

MC3T3-E1 osteoblast precursor cells were stably transfected with a pLuc-*Fgf23* promoter plasmid carrying a secreted luciferase expression cassette under the control of the proximal *Fgf23* promoter and a secreted alkaline phosphatase (SEALP) under the control of a CMV promoter as described previously^53^. Cells were grown for 14 days in osteoblastic medium^54^ and then stimulated with TGF-β ± LY2157299 (10 μM) for 24 h.

### Bronchoalveolar lavage (BAL) in mice and rats

Lung lavage was obtained following established protocols of the lab^55^. Briefly, a tracheal cannula was inserted and the BAL procedure was performed under direct visualization of lung distension (maximum 2 ml), as previously described^56,57^. Cells were pelleted by centrifugation (500–1,100 × *g* for 5 min at 4 °C) and resuspended in 100–150 μl PBS for total cell count determination. Cytospin preparations were stained with modified Wright-Giemsa staining for differential cell counts. For the experiments using CFTR knockout rats^58^ and their control littermates ± pseudomonas infection, we followed previously published protocols^27^.

### ELISA

An ultrasensitive IL-8 enzyme-linked immunosorbent assay (ELISA) from Invitrogen (Thermo Fisher, Waltham, MA, USA) was used. CF-HBEC were stimulated with TGF-β (10 ng/mL) ± FGF23 (25 ng/mL) for 24 hours and 100 μl of the basolateral medium (diluted 1:100 in cell culture medium) was used for measurements. For assessment of IL-6 levels (due to mice not expressing IL-8) from the basolateral medium of MTEC, an IL-6 ELISA kit from Invitrogen (Thermo Fisher, Waltham, MA, USA) was used as per supplier’s instructions. Plasma was used to measure intact FGF23 levels and KL levels in pg/ml using commercially available ELISA kit (FGF23; Immutopics, Clemente, CA, USA and for KL; IBL International, Hamburg, Germany).

### Murine kl overexpression using a lentiviral expression system

Dr. Kuro-o kindly provided us with the full length murine α-klotho^17^, which was cloned into a p38 plasmid containing puromycin resistance. Lentiviral infection of normal HBEC was done before differentiation as previously described^52,59^.

### RNA Extraction and Quantitative RT-PCR

Total RNA was extracted from ALI-cultured human airway epithelial cells using the RNeasy Protect mini kit (Qiagen, Valencia, CA, USA). Reverse transcription was done using the iScript cDNA synthesis kit (Bio-Rad, Hercules, CA, USA) with 1 μg of RNA according to the instructions of the manufacturer. Real-time quantitative PCR was performed using the following TaqMan probes: Hs00241111_m1 for FGFR1, Hs01106910_g1 for FGFR4, Hs00934627_m1 for KL, Hs00174103_m1 for IL-8, Mm00446190_m1 for IL-6, and Hs02758991_g1 for GAPDH.

### Western Blotting

ALI-cultured human airway epithelial cells were lysed in radioimmune precipitation assay buffer containing protease inhibitors. Protein yield was measured by BCA assay (Pierce). Proteins were separated on a 4–20% precast Ready Gel (Bio-Rad) and blotted onto Immobilon-P membranes (Millipore, Billerica, MA). Membranes were blocked with 5% nonfat dry milk in Tris-buffered saline (pH 7.4) with 0.05% Tween 20 (TTBS) for 1 h. Primary antibodies were as follows: rabbit total and phosphor-ERK1/2 (Cell Signaling); rabbit anti-FGFR4 (Santa Cruz) and mouse anti-β-actin (Sigma) antibody. Secondary antibody was an anti-rabbit (Seracare, Milford, MA, USA) or anti-mouse (Seracare, Milford, MA, USA) horseradish peroxidase-linked antibody used at 1:5000 in TTBS for 1 h at room temperature. Positive signals were visualized by chemiluminescence on a ChemiDoc XRS system (Bio-Rad, Hercules, CA, USA). Images were acquired using Image Lab software (Bio-Rad, Hercules, CA, USA).

### Statistics

Experimental data were analyzed with Prism5 (GraphPad Software, Inc., La Jolla, CA) as previously described^38^ using Student’s *t* test and analysis of variance or Kruskal Wallis test with appropriate post tests for at least three independent experiments from each lung and 3–4 different CF lungs. Due to the individual variability of each lung, most changes were normalized to the control and demonstrated in fold changes. Significance was accepted at *p* < 0.05. Linear regression analysis was used for assessment of correlation between FGF23 and KL levels in CF patients. Two outliers were removed using the ROUT method that would have made a linear regression positive; however, statistical analysis using runs testing showed that including them made linear regression analysis inappropriate.

## Electronic supplementary material

Supplemental Figures
