# Supplementary material for: Klotho Inhibits Interleukin-8 Secretion from Cystic Fibrosis Airway Epithelia
Source: Sci Rep. 2017 Oct 30;7:14388. doi: 10.1038/s41598-017-14811-0 (PMC5662572; doi:10.1038/s41598-017-14811-0)

Klotho Inhibits Interleukin-8 Secretion from Cystic Fibrosis Airway Epithelia

*Stefanie Krick[1,2], Nathalie Baumlin[1], Sheyla Paredes Aller[1], Carolina Aguiar[1], Alexander Grabner[3], Juliette Sailland[1], Eliana Mendes[1], Andreas Schmid[1], Lixin Qi[4], Nicolae V David[4], Patrick Geraghty[5], Gwendalyn King[6], Susan E Birket[2], Steven M Rowe[2], Christian Faul[7] and Matthias Salathe[1].

Supplementary Figure 1:
Immunoblot analysis of KL protein levels from CF-HBECs (representative blot from 3 different CF lungs), treated with either vehicle or TGF-β for 24 hours.

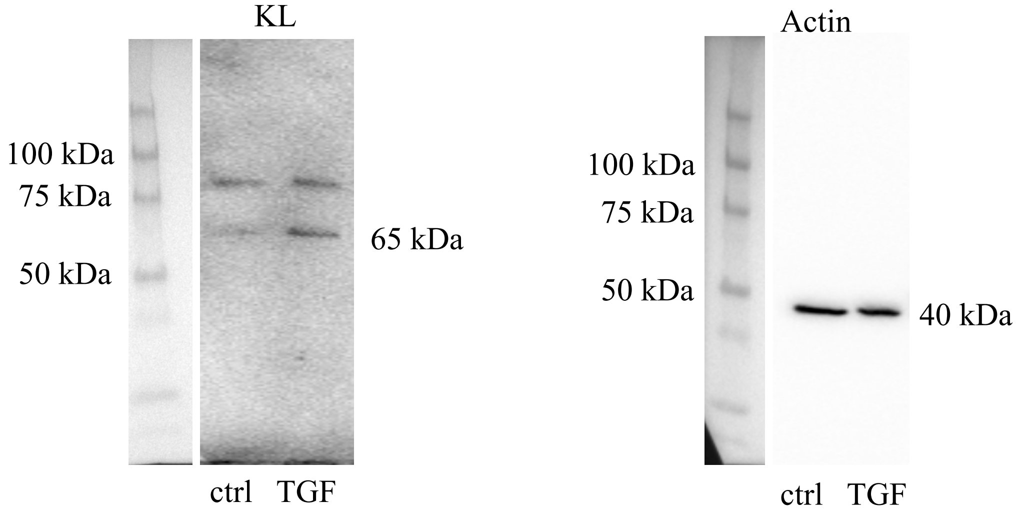

Klotho Inhibits Interleukin-8 Secretion from Cystic Fibrosis Airway Epithelia

*Stefanie Krick[1,2], Nathalie Baumlin[1], Sheyla Paredes Aller[1], Carolina Aguiar[1], Alexander Grabner[3], Juliette Sailland[1], Eliana Mendes[1], Andreas Schmid[1], Lixin Qi[4], Nicolae V David[4], Patrick Geraghty[5], Gwendalyn King[6], Susan E Birket[2], Steven M Rowe[2], Christian Faul[7] and Matthias Salathe[1].

Supplementary Figure 2:
Representative immunoblots are shown after stimulation of CF-HBEC with TGF-β (30 min, 10 ng/ml): obviously increased Smad 3 and minimal increased ERK phosphorylation were seen without changes in PLCγ phosphorylation.

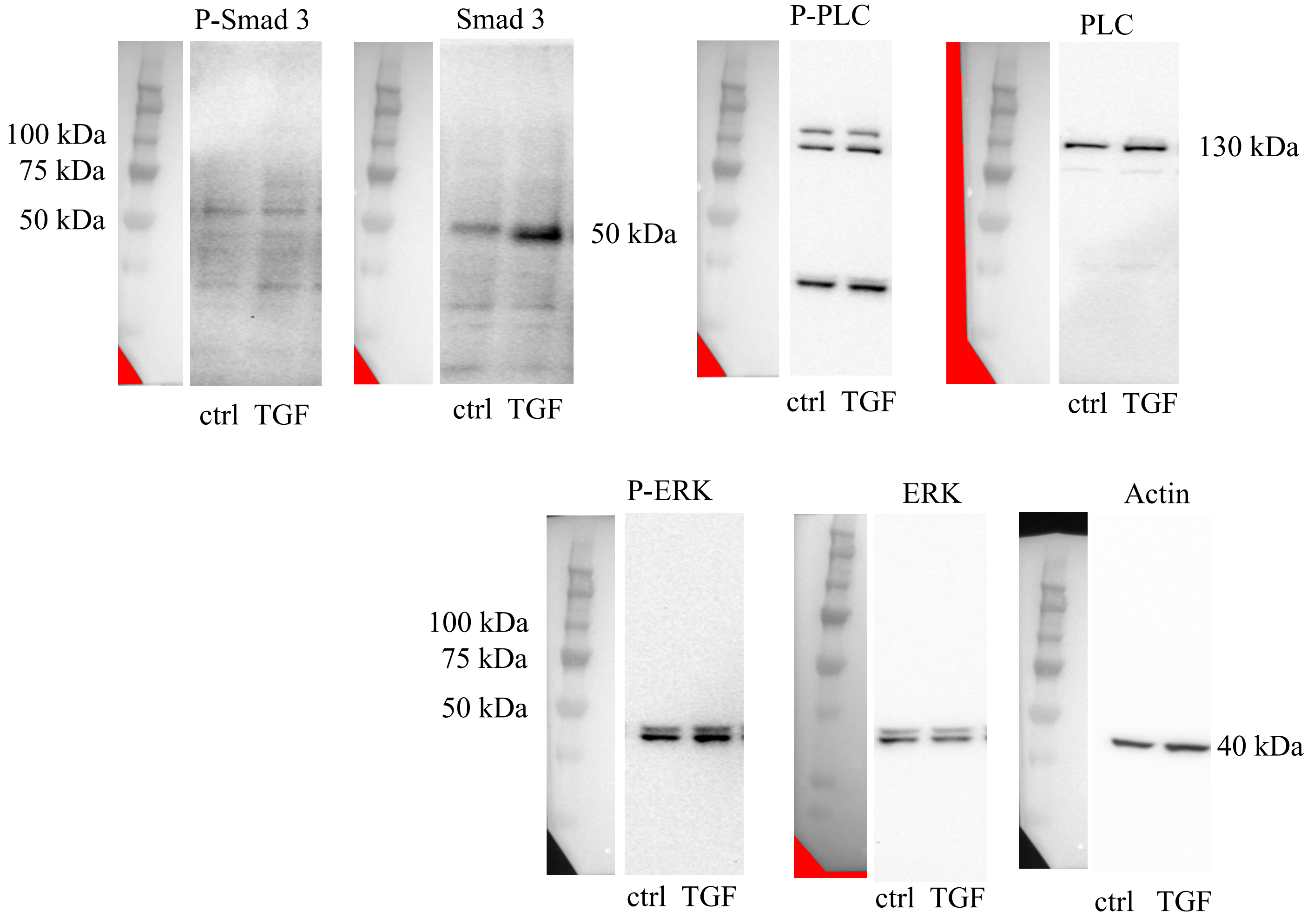

Klotho Inhibits Interleukin-8 Secretion from Cystic Fibrosis Airway Epithelia

*Stefanie Krick[1,2], Nathalie Baumlin[1], Sheyla Paredes Aller[1], Carolina Aguiar[1], Alexander Grabner[3], Juliette Sailland[1], Eliana Mendes[1], Andreas Schmid[1], Lixin Qi[4], Nicolae V David[4], Patrick Geraghty[5], Gwendalyn King[6], Susan E Birket[2], Steven M Rowe[2], Christian Faul[7] and Matthias Salathe[1].

Supplementary Figure 3:
Representative immunoblots showing phospho-ERK, total ERK, phospho-Smad 3 and total Smad 3 in lysates from CF-HBECs after stimulation with TGF-β + FGF23 (30 min, 10 and 25 ng/mL, respectively) ± preincubation with soluble KL (100 ng/ml).

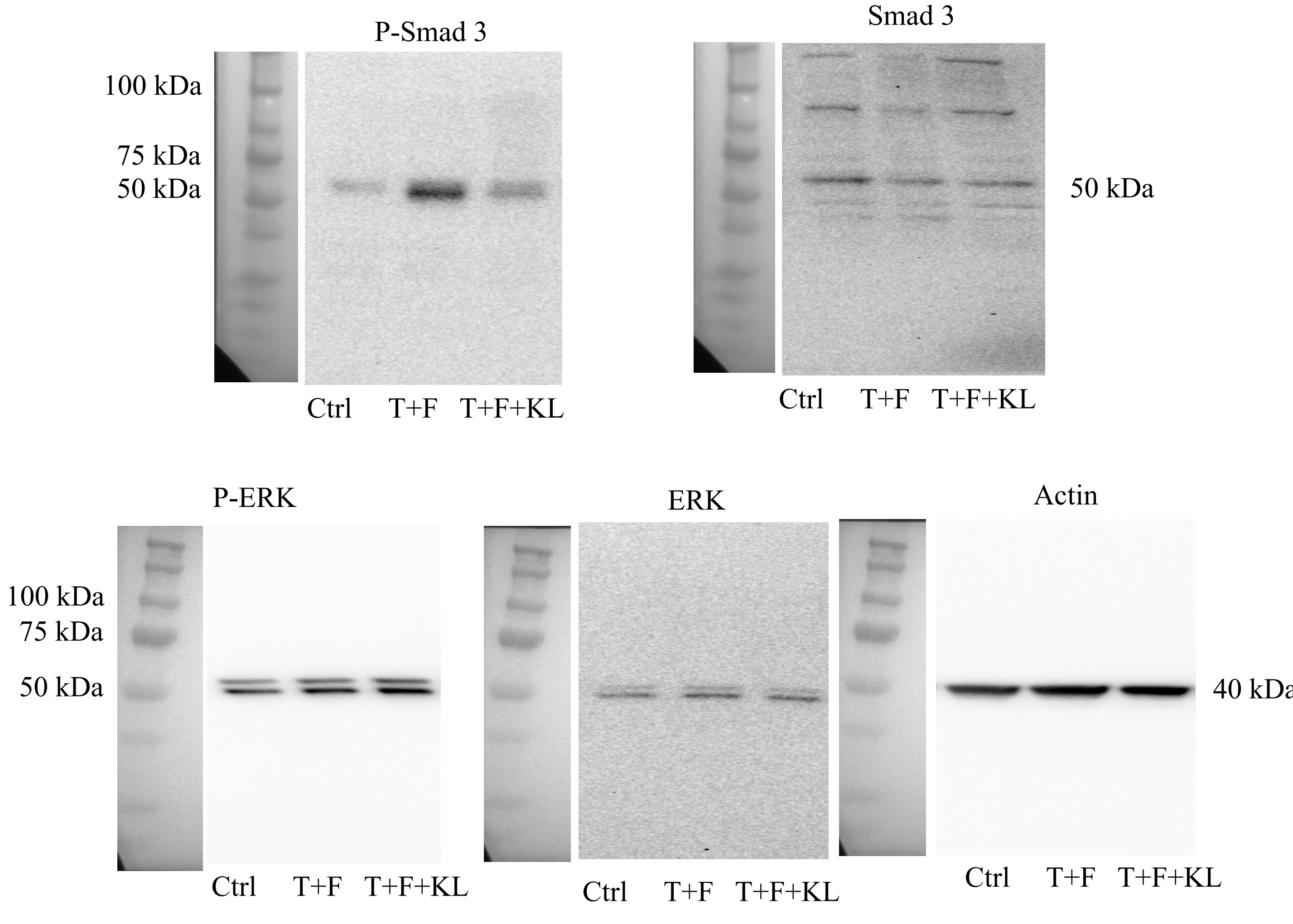

Klotho Inhibits Interleukin-8 Secretion from Cystic Fibrosis Airway Epithelia

*Stefanie Krick[1,2], Nathalie Baumlin[1], Sheyla Paredes Aller[1], Carolina Aguiar[1], Alexander Grabner[3,4], Juliette Sailland[1], Eliana Mendes[1], Andreas Schmid[1], Christian Faul[3,5] and Matthias Salathe[1].

Supplementary Figure 4:
a) Bar graphs indicating relative transcript numbers of klotho mRNA levels in CF-HBEC, treated with CFTR inhibitor, diluted in DMSO (10 mM) for 24 hours. **b)** Bar graphs showing relative transcript number of IL-8 mRNA, normalized to GAPDH in cultures from 5 nonsmokers and 4 CF patients. **c)** Dot blot indicating total neutrophil count from BALF fluid of 4 $kl^{+/+}$ in comparison to 4 $kl^{-/-}$ mice. d) Dot blots indicating KC (Cxcl1) and e) Mip-2 (Cxcl2) mRNA levels normalized to GAPDH in $kl^{+/+}$ compared to $kl^{-/-}$ mice (4 animals per group). (Experiments showing means ± S.E. with *P<0.05 and ***P<0.005).

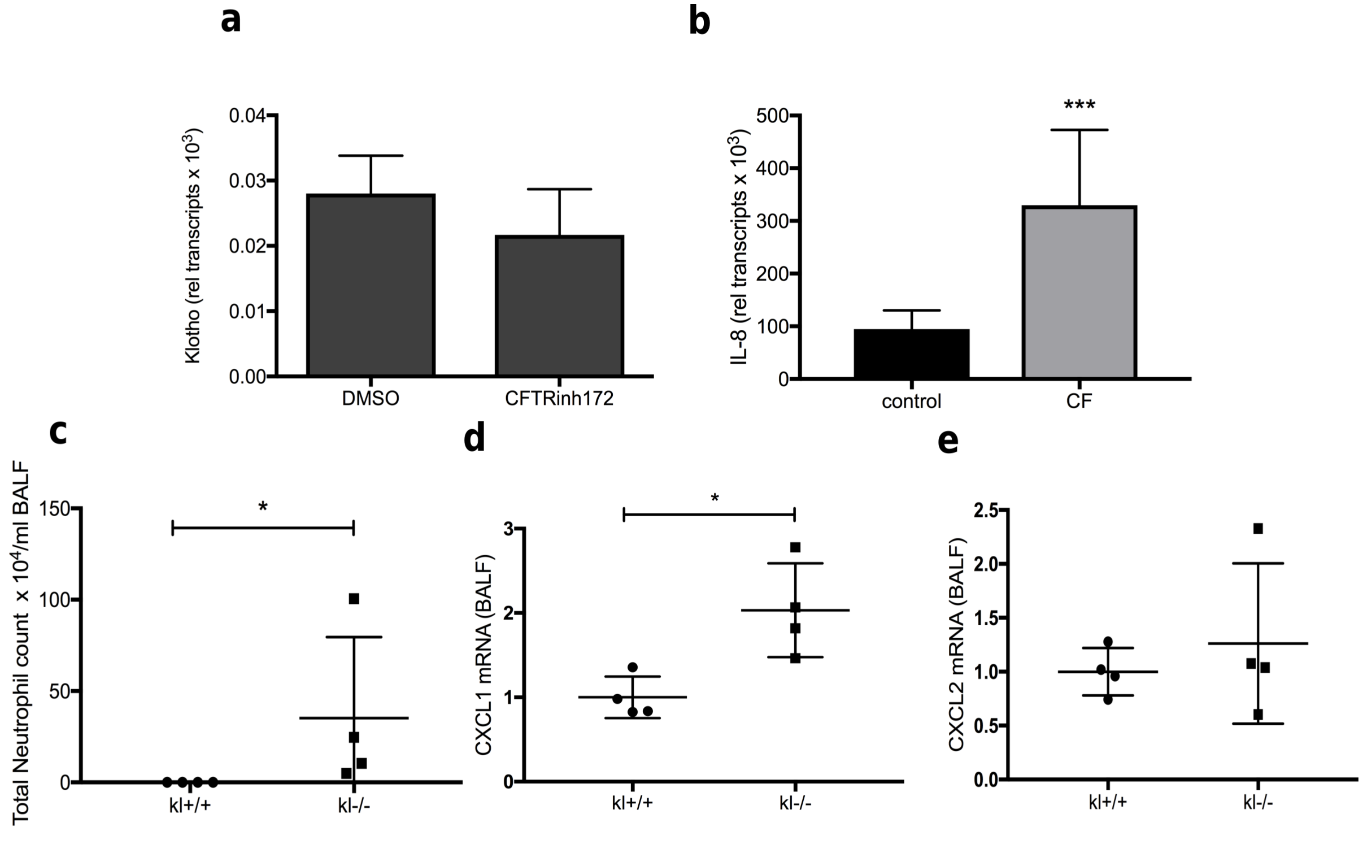

Supplement: Supplementary file 1 — Supplemental Figures [file 41598_2017_14811_MOESM1_ESM.pdf]
